# Inflammation-Driven Epithelial Plasticity in Oral Mucosa Adjacent to Long-Term Restorative Materials: A Retrospective Histopathological and Immunohistochemical Study

**DOI:** 10.3390/medicina62071395

**Published:** 2026-07-18

**Authors:** Roxana-Cristina Mehedinti, Kamel Earar, Ada Stefanescu, Cristina-Mihaela Popescu, Mădălina Nicoleta Matei, Cristian Petcu, Gabriel Valeriu Popa, Dana Tutunaru

**Affiliations:** 1 Medical and Pharmaceutical Research Center, Faculty of Medicine and Pharmacy, “Dunărea de Jos” University of Galati, 800008 Galati, Romania; roxana.mehedinti@ugal.ro (R.-C.M.); kamel.earar@ugal.ro (K.E.); cristina.popescu@ugal.ro (C.-M.P.); madalina.matei@ugal.ro (M.N.M.); dana.tutunaru@ugal.ro (D.T.); 2 Dental-Medicine Department, Faculty of Medicine and Pharmacy, “Dunărea de Jos” University of Galati, 800201 Galati, Romania; 3 ”Sf. Ioan” Clinical Emergency Pediatric Hospital in Galati, 800487 Galati, Romania; 4 Clinical County Emergency Hospital, 010024 Craiova, Romania; cristipetcu80@yahoo.com; 5 Clinical County Emergency Hospital “Sfantul Apostol Andrei”, 800578 Galati, Romania

**Keywords:** CK19, COX-2, epithelial plasticity, epithelial remodeling, Ki67, immunohistochemistry, oral mucosa, restorative materials

## Abstract

*Background and Objectives*: Long-standing contact between oral mucosa and restorative materials may be associated with adaptive epithelial and stromal remodeling. This study evaluated non-dysplastic epithelial plasticity in the oral mucosa adjacent to long-term restorative materials. *Materials and Methods:* This retrospective study included 150 formalin-fixed, paraffin-embedded oral mucosal specimens divided into five groups: control, dental amalgam, methacrylate-based resin composite, clinically documented all-ceramic restorations, and metallic/metal–ceramic restorations (*n* = 30 each). Exposed cases had documented mucosal adjacency or repetitive contact with a dominant restorative material for at least 5 years. Histopathological parameters, CK19, Ki67, p53, and COX-2 expression were assessed, and MAERS was calculated. Statistical significance was set at *p* < 0.05. *Results:* MAERS differed significantly among groups (*p* < 0.001), with the highest mean values in metallic/metal–ceramic specimens (10.3 ± 2.4) and dental amalgam specimens (9.7 ± 2.1), intermediate values in resin composite specimens (7.1 ± 1.8), and the lowest values in all-ceramic (5.0 ± 1.4) and control specimens (4.8 ± 1.3). Suprabasal CK19 redistribution was observed in 22/30 metallic/metal–ceramic specimens (73.3%) and 20/30 amalgam specimens (66.7%), elevated Ki67 expression in 22/30 (73.3%) and 19/30 (63.3%), and moderate-to-strong COX-2 expression in 21/30 (70.0%) and 19/30 (63.3%), respectively. p53 expression was heterogeneous and did not show dominant, strong, or diffuse overexpression. *Conclusions:* Metallic/metal–ceramic restorations showed the highest remodeling burden, dental amalgam showed a similarly high profile, resin composite showed an intermediate profile, and all-ceramic-associated mucosa remained close to control specimens. These findings suggest material-dependent, inflammation-associated epithelial plasticity without evidence of epithelial dysplasia.

## 1. Introduction

Dental restorative materials remain in prolonged contact with oral tissues and therefore represent a clinically relevant interface between biomaterials and the stratified squamous epithelium of the gingival and alveolar mucosa. Although contemporary restorative dentistry prioritizes mechanical durability, marginal integrity, esthetics, and biocompatibility, the long-term biological response of adjacent mucosa is not uniform across material classes. Metallic alloys, metal–ceramic systems, dental amalgam, resin composites, and ceramic restorations differ in surface chemistry, corrosion behavior, ion release, roughness, plaque-retentive potential, and potential to favor local inflammatory microenvironments [1,2,3]. These features may influence epithelial homeostasis, not through a direct deterministic pathway but through chronic low-grade stimulation at the material–mucosa interface [1,2,3,4].

The oral mucosa is a dynamic barrier tissue in which epithelial maturation, basal cell renewal, stromal remodeling, and immune surveillance are tightly coordinated [5,6]. Persistent mechanical irritation, microbial biofilm accumulation, and exposure to degradation products or released ions can be associated with adaptive epithelial and stromal responses, including basal/parabasal compartment expansion, epithelial thickening, inflammatory infiltration, and subepithelial fibrosis [5,6,7]. In the context of long-standing contact with dental restorative materials, such responses may overlap with reactive or lichenoid mucosal patterns rather than necessarily indicating epithelial dysplasia [8,9,10]. However, their biological interpretation becomes more complex when structural changes are accompanied by shifts in epithelial immunophenotype, proliferative activity, and inflammatory signaling [6,8,9,10]. For this reason, histopathological assessment alone may underestimate the extent of material-associated mucosal adaptation.

In this context, epithelial homeostasis refers to the dynamic balance that preserves oral epithelial integrity through coordinated basal cell renewal, differentiation, maturation, barrier function, stromal–epithelial signaling, and immune surveillance [5,6,7]. Persistent local stimulation may disturb this balance and induce reactive, non-dysplastic epithelial remodeling, including basal/parabasal expansion, acanthosis, altered keratin expression, increased proliferative activity, and inflammation-associated stromal changes [5,6,7,8,9,10]. These changes should be distinguished from epithelial dysplasia, which denotes potentially premalignant architectural and cytological abnormalities such as disturbed stratification, loss of maturation, cellular atypia, abnormal mitotic activity, and loss of polarity [8,9,10].

Immunohistochemical markers can provide additional insight into inflammation-associated epithelial plasticity [11,12,13]. Cytokeratin 19 (CK19) is generally limited in architecturally preserved oral epithelium, whereas suprabasal redistribution may indicate altered epithelial differentiation or expansion of a less mature epithelial compartment [11]. Ki67 reflects proliferative activity and helps identify enlargement of the cycling basal/parabasal cell population [11,12,13,14]. Cyclooxygenase-2 (COX-2) is involved in inflammatory signaling and may link stromal inflammation to epithelial response patterns [12,13]. By contrast, p53, a nuclear tumor suppressor protein encoded by the TP53 gene, must be interpreted cautiously when assessed by immunohistochemistry. In non-dysplastic mucosa, limited or heterogeneous nuclear p53 staining is more appropriately considered a marker of cellular stress response rather than evidence of malignant transformation or TP53 mutation [11,13,15].

Despite the extensive use of restorative materials, comparative human studies evaluating material-associated oral mucosal changes across multiple restorative categories remain limited [16,17,18,19]. Most available evidence addresses isolated material reactions, contact lesions, hypersensitivity phenomena, lichenoid mucosal conditions, or associations between dental restorations/prostheses and oral mucosal disorders [16,17,18,19]. In addition, distinguishing histopathologically between reactive, lichenoid, and potentially dysplastic epithelial alterations can be challenging and often requires careful clinicopathological correlation [20,21]. This gap is relevant because material-related mucosal changes may be subtle, multifactorial, and dependent on interactions among biomaterial properties, local inflammation, epithelial turnover, and stromal remodeling.

Given these limitations, the use of an integrated remodeling score is relevant because a single histopathological parameter or immunohistochemical marker may not fully capture material-associated mucosal changes. The Material-Associated Epithelial Remodeling Score (MAERS) was therefore used as an exploratory composite to summarize the cumulative burden of structural epithelial remodeling, inflammatory infiltration, proliferative activity, altered epithelial differentiation, and inflammatory signaling within a single analytical framework. This approach is important in the current context of long-term restorative material use because subtle non-dysplastic mucosal adaptations may remain clinically underrecognized but may provide insight into the biological interaction between restorative materials and adjacent oral tissues.

Therefore, this retrospective histopathological and immunohistochemical study evaluated oral mucosal specimens adjacent to long-standing restorative materials, including dental amalgam, resin composite, ceramic, and metallic/metal–ceramic restorations, compared with control mucosa. The study aimed to characterize material-associated epithelial and stromal remodeling patterns using conventional histopathological parameters and the expression of CK19, Ki67, p53, and COX-2. MAERS was additionally used to quantify cumulative remodeling burden. The null hypothesis was that long-term mucosal adjacency to different restorative material classes would not be associated with significant differences in histopathological remodeling, immunohistochemical expression patterns, or cumulative MAERS values. The alternative study hypothesis was that different restorative material categories would be associated with distinct, non-dysplastic patterns of inflammation-driven epithelial plasticity and remodeling burden.

## 2. Materials and Methods

### 2.1. Study Design and Ethical Approval

This was a retrospective cross-sectional comparative archival study. It was conducted in accordance with the Declaration of Helsinki. It was approved by the Ethics Committee of the University Center of Dental Medicine of Galati, “Dunărea de Jos” University of Galati, Romania (approval no. 16/23 April 2026).

All specimens were obtained during routine clinical care, independent of and before study conception, and no prospective specimen collection, clinical intervention, or patient recruitment was performed for the present study. Therefore, ethical approval was obtained before retrospective identification, extraction, and analysis of the archived material, consistent with institutional and national requirements governing retrospective research on archived clinical specimens.

The formalin-fixed, paraffin-embedded oral mucosal specimens were obtained during alveoloplastic extraction procedures performed between April 2022 and May 2024 in the affiliated dental clinical setting. Storage specimens and all histopathological and immunohistochemical analyses were performed at the Department of Oral Pathology at the same institution.

### 2.2. Experimental Design and Study Workflow

This retrospective, cross-sectional, comparative study evaluated archived oral mucosal specimens stratified by restorative material exposure category. Restorative material category served as the primary exposure variable, with histopathological remodeling parameters, immunohistochemical marker expression (CK19, Ki67, p53, COX-2), and MAERS as outcome variables, and age and sex as covariates. Eligible specimens, selected according to the criteria detailed in Section 2.3, were allocated into five comparison groups (Section 2.4) and underwent histopathological and immunohistochemical assessment (Section 2.5, Section 2.6, Section 2.7 and Section 2.8), followed by intergroup statistical comparison (Section 2.9).

### 2.3. Inclusion and Exclusion Criteria

Inclusion criteria comprised oral mucosal tissue obtained during alveoloplastic extraction procedures, histologically preserved non-neoplastic stratified squamous epithelium, and sufficient tissue available for complete histopathological and immunohistochemical assessment. For the four exposed groups, inclusion additionally required documented long-term contact between the oral mucosa and a single dominant restorative material; for the control group, inclusion required documented absence of direct mucosal contact with restorative materials at the sampled site.

Exclusion criteria included previously diagnosed oral epithelial dysplasia or oral squamous cell carcinoma, autoimmune mucosal disease, active periodontal disease, extensive ulceration or severe tissue fragmentation, inadequate tissue preservation, incomplete clinical documentation, and cases with multiple adjacent restorative materials preventing reliable exposure classification. Cases with documented suppuration, acute periodontal inflammation, or severe local periodontal destruction at the sampled site were also excluded.

Clinical records were additionally reviewed, when available, for relevant patient-related inflammatory modifiers, including diabetes mellitus, systemic inflammatory or autoimmune diseases, chronic medication use, smoking status, alcohol consumption, and periodontal health status. Incomplete documentation of lifestyle-related and detailed periodontal variables was acknowledged as a limitation of the retrospective design.

### 2.4. Study Cohort and Group Allocation

Cohort assembly proceeded by systematically applying predefined eligibility criteria to the archived specimen pool. Archived formalin-fixed, paraffin-embedded oral mucosal specimens retrieved from the Department of Oral Pathology were screened according to the criteria detailed in Section 2. Each eligible patient contributed a single specimen assigned to one exposure category based on the dominant restorative material in direct contact with, or close anatomical proximity to, the sampled mucosa. Assignment to an exposed group required clinical documentation of long-standing mucosal adjacency, repetitive contact, or direct contact with a single dominant restorative material for at least 5 years. The control group comprised specimens obtained from comparable alveoloplastic extraction procedures in patients with documented absence of direct mucosal contact with restorative materials at the sampled site, providing an unexposed reference category against which material-specific remodeling could be evaluated.

Five clinically defined study groups were established: control (*n* = 30), dental amalgam (*n* = 30), resin composite (*n* = 30), ceramic restoration (*n* = 30), and metallic/metal–ceramic restoration (*n* = 30). The final analytic cohort comprised 150 patients and 150 corresponding specimens. The ceramic restoration group encompassed clinically documented all-ceramic restorations without a metallic substructure; where available, archival records indicated zirconia-based or lithium–disilicate composition, though the absence of consistent manufacturer-level documentation across cases precluded subclassification, and the group was therefore analyzed as a single all-ceramic category. Restoration age, restoration integrity, and the nature of the mucosal-material relationship (direct, repetitive, or close anatomical proximity) were reviewed when available. However, direct instrumented assessment of surface corrosion, wear, roughness, or marginal degradation was not consistently documented and was accordingly not analyzed as an independent variable.

Application of the eligibility criteria identified eligible archived cases across the five restorative material categories, with 30–34 cases per group. The smallest eligible category comprised 30 cases; this number was therefore adopted as the standardized group size. In categories where eligible cases exceeded 30, the final 30 specimens were selected by random sampling from the eligible pool. Groups were additionally balanced by age, sex, anatomical sampling site, and duration of material contact, where archival documentation permitted.

This sample size was additionally compatible with the logistical demands of paired histopathological scoring and four-marker immunohistochemical evaluation across 150 specimens by two independently blinded observers, a consideration of particular relevance given the labor-intensive nature of dual-observer semi-quantitative assessment.

As a secondary, post hoc observation, power analysis based on the observed effect size for the primary outcome measure (Cohen’s f = 1.24 for intergroup MAERS differences) indicated that the achieved sample size provided power exceeding 0.99 at α = 0.05. Since this estimate is derived from the same dataset used to generate the reported effects, it is presented as a descriptive characterization of the achieved cohort rather than an independent, a priori justification of the sample size, consistent with established methodological caveats regarding power analyses.

**Figure 1:**
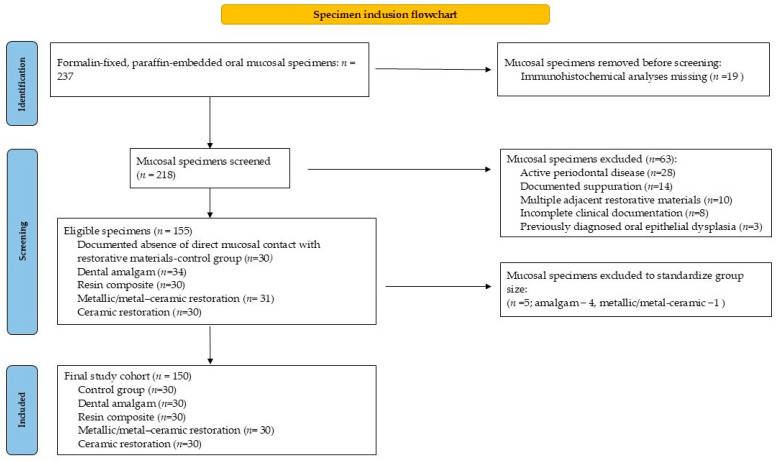
Flowchart of case selection and group allocation.

The full case selection process, including the number of specimens initially retrieved, reasons for exclusion at each stage, and final group allocation, is illustrated in Figure 1.

### 2.5. Histopathological Evaluation

Paraffin blocks were sectioned at 4 μm and stained with hematoxylin–eosin (H&E; Bio-Optica, Milan, Italy) for histopathological assessment. Two experienced oral pathologists, blinded to restorative material category, scored all slides independently before consensus; interobserver agreement was substantial (Cohen’s κ = 0.82), and discordant assessments were resolved by joint re-evaluation.

Epithelial and stromal remodeling was captured with a predefined semi-quantitative system. The assessed parameters and their grading criteria are summarized in Table 1.

Microscopic evaluation emphasized epithelial architectural integrity, maturation pattern, basal/parabasal compartment expansion, the epithelial–stromal interface, and any features suggestive of dysplasia. Specimens with architectural or cytological changes compatible with oral epithelial dysplasia were excluded from the comparative analysis.

### 2.6. Immunohistochemistry

The 4 μm sections underwent deparaffinization in xylene (Merck KGaA, Darmstadt, Germany) and rehydration through graded alcohols (Merck KGaA, Darmstadt, Germany). They were subsequently mounted on positively charged slides (Thermo Fisher Scientific, Waltham, MA, USA), and heat-induced epitope retrieval was performed. This was an extension of the protocol established in the authors’ prior single-material study to which COX-2 immunostaining was added [11]. The primary antibodies, clones, retrieval buffers, and suppliers are summarized in Table 2.

Detection used a polymer-based peroxidase system (EnVision FLEX Detection System; Dako, Glostrup, Denmark) with 3,3′-diaminobenzidine (DAB; Dako, Glostrup, Denmark) as chromogen. Hematoxylin counterstaining (Merck KGaA, Darmstadt, Germany), dehydration, clearing, and mounting with DPX medium (Sigma-Aldrich, St. Louis, MO, USA) were the next steps. Positive and negative tissue controls were included in each immunohistochemical run.

### 2.7. Immunohistochemical Scoring

Two calibrated observers, blinded to restorative material category, independently scored immunohistochemical expression semi-quantitatively; agreement was substantial (κ = 0.86), with discordant scores resolved by joint review and consensus.

CK19 and p53 scoring followed the criteria established in the authors’ prior study [11]; the Ki-67 labeling index was derived from at least 300 epithelial cells counted within the most proliferative fields (“hot spots”). COX-2, introduced specifically for this comparative design, was scored according to combined epithelial and subepithelial staining intensity and distribution. Score definitions for all four markers are summarized in Table 3.

Because no case showed dysplastic or malignant morphology, p53 positivity was interpreted as a marker of cellular stress rather than as an indicator of TP53 mutational status.

### 2.8. MAERS

To evaluate the cumulative burden of structural, inflammatory, and immunophenotypic remodeling at the individual case level, MAERS was constructed specifically for the present study. It has not been previously validated as a standardized clinical or histopathological scoring system. Therefore, it was used as an exploratory composite index intended to summarize the cumulative burden of inflammation-associated epithelial remodeling. Internal consistency of the five MAERS components was assessed using Cronbach’s alpha, which indicated good reliability (α = 0.84).

The conceptual framework of MAERS builds on the exploratory Integrated Epithelial Remodeling Score previously described by Mehedinti et al. [11]; however, the present composite score differs in component parameters, incorporating basal hyperplasia and inflammatory infiltrate intensity as structural anchors and substituting COX-2 expression for p53. This compositional difference reflects the broader multi-material comparative design of the present study, in which COX-2 was selected as a primary inflammatory signaling marker, and p53 was retained as a separate, independently interpreted stress-response variable rather than a component of the coordinated remodeling phenotype.

The score was calculated as the sum of five parameters: basal hyperplasia, intensity of the inflammatory infiltrate, CK19 redistribution, categorized Ki67 proliferative activity, and COX-2 expression.

The theoretical MAERS range was 1–14, with higher values indicating greater epithelial remodeling intensity and inflammation-associated epithelial plasticity. p53 expression was not included in the composite score because it was considered a marker of heterogeneous cellular stress response rather than a primary component of the coordinated remodeling phenotype.

MAERS was analyzed as a continuous variable and was used to compare the cumulative remodeling burden among the control, dental amalgam, resin composite, ceramic, and metallic/metal-ceramic groups.

### 2.9. Statistical Analysis

Analyses were performed in IBM SPSS Statistics v26.0 (IBM Corp., Armonk, NY, USA) and GraphPad Prism v9.0 (GraphPad Software, San Diego, CA, USA).

Continuous variables are reported as mean ± SD and ordinal variables as median with interquartile range. Normality was evaluated with the Shapiro–Wilk test and visual inspection of distribution plots. Both the histopathological and immunohistochemical scores are ordinal by design, and they were analyzed nonparametrically regardless of distributional findings.

Continuous variables were compared across groups using one-way ANOVA or the Kruskal–Wallis test, depending on distributional characteristics. The Kruskal–Wallis test was used when comparing ordinal histopathological and immunohistochemical scores. Significant omnibus results were followed by Dunn’s post hoc pairwise testing with Bonferroni correction. Categorical variables were compared using the chi-square test or Fisher’s exact test, as appropriate.

Spearman’s rank correlation coefficient was used to evaluate associations among age, sex, restorative material category, histopathological parameters, immunohistochemical markers, and MAERS.

To account for potential confounding by age and sex, the association between restorative material category and MAERS was further assessed using multivariable linear regression adjusted for these covariates. For the principal high-grade remodeling outcomes (inflammatory infiltrate ≥2, basal hyperplasia ≥2, suprabasal CK19 expression ≥2, Ki67 score 2, and COX-2 score ≥2), binary logistic regression models entered restorative material category, age, and sex simultaneously as predictors. Statistical significance was defined as a two-tailed *p* < 0.05.

## 3. Results

### 3.1. Study Cohort and Clinicodemographic Characteristics

A total of 150 unique patients were included in the analysis, corresponding to 150 formalin-fixed, paraffin-embedded oral mucosal specimens. The overall mean age of the cohort was 50.4 ± 8.6 years. The study population included 86 female patients (57.3%) and 64 male patients (42.7%). The cohort was evenly distributed across five groups based on the dominant restorative material adjacent to the sampled mucosa: control, dental amalgam, resin composite, ceramic, and metallic/metal–ceramic, with 30 specimens per group.

All specimens consisted of gingival or alveolar mucosal tissue obtained during alveoloplastic extraction procedures. No statistically significant intergroup differences were observed for age (*p* = 0.412), sex distribution (*p* = 0.962), or anatomical sampling site (*p* = 0.973). In the exposed groups, mean contact duration ranged from 10.7 ± 4.2 years in the ceramic group to 13.4 ± 5.7 years in the metallic/metal–ceramic group, without statistically significant differences between groups (*p* = 0.286). All exposed specimens had documented contact duration of at least 5 years. The clinicodemographic characteristics of the study cohort are summarized in Table 4.

### 3.2. Histopathological Remodeling Patterns Across Restorative Material Groups

Comparative histopathological evaluation showed significant intergroup differences for all assessed epithelial and stromal parameters. No architectural or cytological features compatible with oral epithelial dysplasia were identified in any study group.

Basal hyperplasia differed significantly among groups (*p* < 0.001). Scores 2–3 were more frequent in the dental amalgam and metallic/metal–ceramic groups than in the control and ceramic groups. Acanthosis also showed significant intergroup variation (*p* = 0.002), with higher scores observed more frequently in the dental amalgam and metallic/metal–ceramic groups.

Parakeratosis was significantly more frequent in the dental amalgam and metallic/metal–ceramic groups than in the control and ceramic groups (*p* = 0.004). Spongiosis also differed significantly among restorative material categories (*p* = 0.006), with moderate-to-marked scores occurring more often in the dental amalgam and metallic/metal–ceramic groups.

Subepithelial fibrosis showed significant intergroup variation (*p* < 0.001). Scores 2–3 were most frequent in the dental amalgam and metallic/metal–ceramic groups, whereas lower fibrosis scores predominated in the control and ceramic groups. Inflammatory infiltrate intensity also differed significantly among groups (*p* < 0.001), with moderate-to-intense inflammatory infiltration occurring most frequently in the dental amalgam and metallic/metal–ceramic groups.

Overall, the control and ceramic groups showed lower histopathological scores across most parameters, resin composite specimens showed intermediate values, and dental amalgam and metallic/metal–ceramic specimens showed the highest frequencies of high-grade remodeling parameters. The distribution of histopathological parameters according to restorative material category is summarized in Table 5.

**Figure 2:**
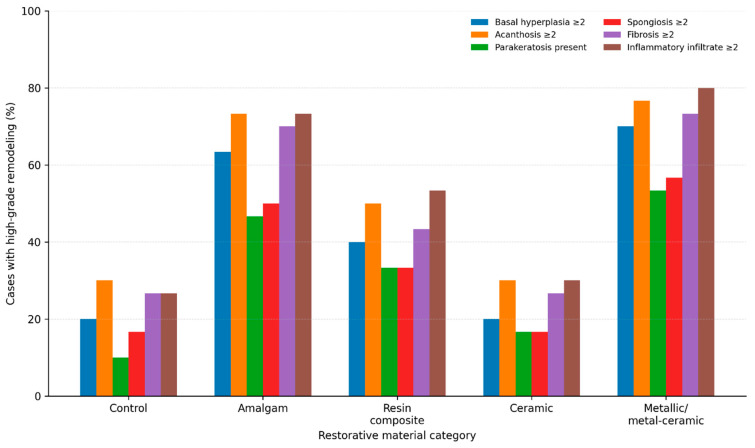
Distribution of high-grade histopathological remodeling parameters according to restorative material category.

The percentage of cases showing high-grade histopathological remodeling is illustrated in Figure 2. High-grade basal hyperplasia, acanthosis, spongiosis, subepithelial fibrosis, and an inflammatory infiltrate were more frequent in the metallic/metal–ceramic and dental amalgam groups, intermediate in the resin composite group, and less frequent in the ceramic and control groups.

### 3.3. Immunohistochemical Expression Patterns

Immunohistochemical evaluation demonstrated significant intergroup differences for CK19, Ki67, p53, and COX-2 expression across restorative material categories.

CK19 expression differed significantly among groups (*p* < 0.001). Suprabasal CK19 redistribution, defined as scores 2–3, was observed in 5/30 control specimens (16.7%) and 5/30 ceramic-associated specimens (16.7%), compared with 12/30 resin composite specimens (40.0%), 20/30 dental amalgam specimens (66.7%), and 22/30 metallic/metal–ceramic specimens (73.3%).

Ki67 proliferative activity also showed significant intergroup variation (*p* < 0.001). Elevated Ki67 expression, defined as score 2, was identified in 4/30 control specimens (13.3%) and 4/30 ceramic-associated specimens (13.3%), compared with 11/30 resin composite specimens (36.7%), 19/30 dental amalgam specimens (63.3%), and 22/30 metallic/metal–ceramic specimens (73.3%).

COX-2 expression differed significantly among groups (*p* < 0.001). Moderate-to-strong COX-2 expression, defined as scores 2–3, was observed in 4/30 control specimens (13.3%) and 4/30 ceramic-associated specimens (13.3%), compared with 10/30 resin composite specimens (33.3%), 19/30 dental amalgam specimens (63.3%), and 21/30 metallic/metal–ceramic specimens (70.0%).

p53 expression showed significant intergroup variation (*p* = 0.031). Score 2 p53 expression was observed in 2/30 control specimens (6.7%), 2/30 ceramic-associated specimens (6.7%), 4/30 resin composite-associated specimens (13.3%), 5/30 dental amalgam-associated specimens (16.7%), and 7/30 metallic/metal–ceramic-associated specimens (23.3%). Strong diffuse p53 overexpression was not observed as a dominant pattern in any group.

Overall, CK19 ≥2, Ki67 score 2, and COX-2 ≥2 were most frequent in the metallic/metal–ceramic and dental amalgam groups, intermediate in the resin composite group, and lowest in the ceramic and control groups. p53 score 2 was less frequent and showed a less pronounced intergroup gradient. The full distribution of immunohistochemical scores according to restorative material category is presented in Table 6.

**Figure 3:**
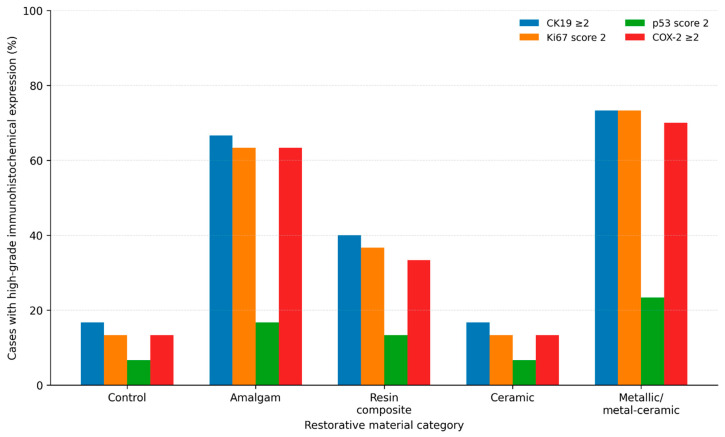
Representative immunohistochemical expression patterns of CK19, Ki67, p53, and COX-2 across restorative material groups.

The percentage of cases showing high-grade immunohistochemical expression is illustrated in Figure 3. CK19 ≥2, Ki67 score 2, and COX-2 ≥2 showed a parallel increase across restorative material groups, with the highest percentages in the metallic/metal–ceramic and dental amalgam groups, intermediate percentages in the resin composite group, and the lowest percentages in the ceramic and control groups. p53 score 2 was less frequent and showed a less pronounced intergroup gradient. This expression pattern was further interpreted in the Section 4 in relation to chronic local inflammation, material degradation, plaque-retentive surface characteristics, and possible metal ion release at the material–mucosa interface.

### 3.4. Comparative Analysis of MAERS Across Restorative Materials

A comparative analysis of the MAERS revealed significant intergroup differences across restorative material categories (*p* < 0.001). The highest MAERS values were observed in the metallic/metal-ceramic and dental amalgam groups, whereas the ceramic and control groups showed the lowest cumulative remodeling burden. Resin composite-associated specimens displayed intermediate MAERS values between the metallic/amalgam groups and the ceramic/control groups.

Mean MAERS values were 4.8 ± 1.3 in the control group, 9.7 ± 2.1 in the dental amalgam group, 7.1 ± 1.8 in the resin composite group, 5.0 ± 1.4 in the ceramic group, and 10.3 ± 2.4 in the metallic/metal-ceramic group. Pairwise post hoc analysis demonstrated significantly higher MAERS values in the metallic/metal-ceramic and amalgam groups compared with the ceramic and control groups (adjusted *p* < 0.01). No statistically significant difference was identified between the control and ceramic groups after Bonferroni correction. This similarity indicates that, in the present cohort, all-ceramic-associated mucosa showed a cumulative remodeling profile close to that of control mucosa, as further interpreted in the Section 4.

The distribution of MAERS values also demonstrated narrower interquartile ranges in the control and ceramic groups and wider score dispersion in the metallic/metal-ceramic and amalgam groups, indicating greater interindividual variability in high-grade remodeling phenotypes associated with chronic exposure to metallic restorative materials.

Ninety-five percent confidence intervals confirmed progressive increases in scores across restorative material categories. The metallic/metal-ceramic group demonstrated the highest cumulative remodeling burden (95% CI: 9.4–11.2), followed by the dental amalgam group (95% CI: 8.9–10.5), resin composite group (95% CI: 6.5–7.7), ceramic group (95% CI: 4.5–5.5), and control group (95% CI: 4.3–5.3).

**Figure 4:**
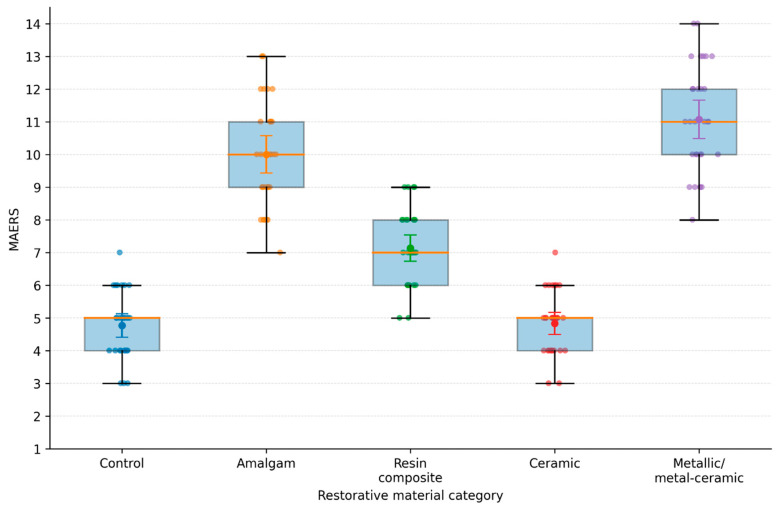
Distribution of MAERS according to restorative material category.

Overall, the MAERS analysis demonstrated a structured material-dependent remodeling gradient characterized by minimal cumulative remodeling in ceramic-associated mucosa, intermediate remodeling in resin composite-associated specimens, and the highest remodeling burden in mucosa adjacent to metallic and dental amalgam restorations. The distribution of MAERS values according to restorative material category is illustrated in Figure 4. Boxplots illustrate the distribution of MAERS values across study groups. Central lines represent median values, boxes indicate interquartile ranges, whiskers represent minimum and maximum values, and dots correspond to individual cases. Colored dots and error bars indicate group mean values with 95% confidence intervals. Colors were used exclusively for visual differentiation between restorative material groups and do not represent additional biological or statistical categories.

### 3.5. Correlation Network of Histopathological and Immunohistochemical Parameters

Spearman correlation analysis demonstrated a structured interaction network linking inflammatory remodeling, epithelial proliferative activity, and immunophenotypic redistribution patterns across the study cohort. The strongest positive correlations were observed between CK19 redistribution and Ki67 proliferative activity (ρ = 0.71, *p* < 0.001) and between COX-2 expression and inflammatory infiltrate intensity (ρ = 0.76, *p* < 0.001). These findings support the presence of a coordinated inflammation-associated epithelial remodeling phenotype.

Moderate positive correlations were also observed between basal hyperplasia and Ki67 expression (ρ = 0.63, *p* < 0.001), basal hyperplasia and CK19 redistribution (ρ = 0.59, *p* < 0.001), and inflammatory infiltrate intensity and subepithelial fibrosis (ρ = 0.68, *p* < 0.001). Together, these associations indicate coupling between chronic inflammatory stimulation, epithelial compartment expansion, and stromal remodeling.

MAERS demonstrated strong positive correlations with CK19 redistribution (ρ = 0.82, *p* < 0.001), Ki67 proliferative activity (ρ = 0.79, *p* < 0.001), inflammatory infiltrate intensity (ρ = 0.84, *p* < 0.001), and COX-2 expression (ρ = 0.81, *p* < 0.001), confirming its ability to reflect cumulative epithelial and stromal remodeling burden across restorative material groups.

In contrast, p53 expression showed weaker and more heterogeneous correlations with the remaining histopathological and immunohistochemical parameters, including weak-to-moderate associations with CK19 redistribution (ρ = 0.31, *p* = 0.004) and Ki67 proliferative activity (ρ = 0.28, *p* = 0.011). These findings support the interpretation of p53 as a variable stress-associated marker rather than a central component of the coordinated remodeling network.

**Figure 5:**
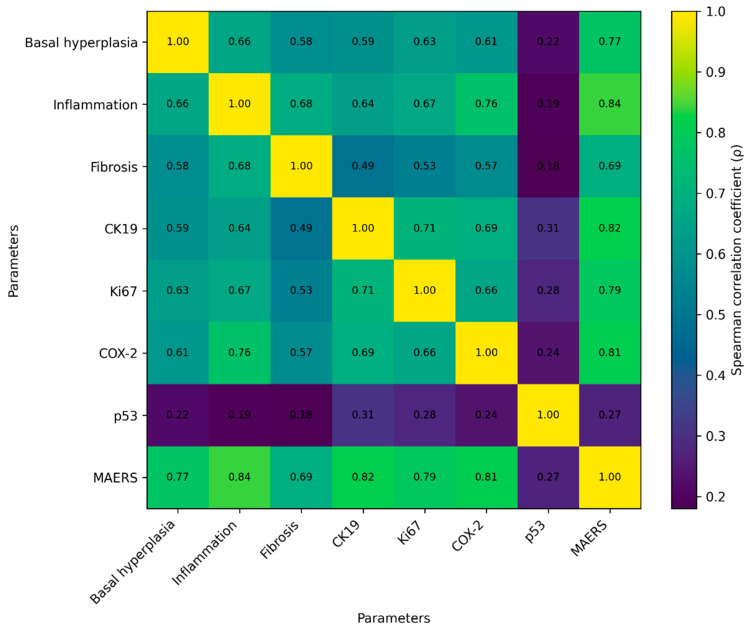
Spearman correlation matrix of histopathological and immunohistochemical remodeling parameters.

Overall, the correlation analysis identified a consistent CK19–Ki67–COX-2 remodeling axis associated with inflammatory epithelial plasticity and cumulative remodeling burden. The complete Spearman correlation matrix is illustrated in Figure 5. The heatmap illustrates the strength of pairwise Spearman correlation coefficients (ρ) among histopathological variables, immunohistochemical markers, and MAERS. Warmer colors indicate stronger positive correlations, while the numerical values within each cell represent individual Spearman correlation coefficients. The strongest associations were observed between inflammatory infiltrate intensity, CK19 redistribution, COX-2 expression, Ki67 proliferative activity, and MAERS, supporting the presence of a coordinated inflammation-associated epithelial remodeling network. The clinical relevance of this correlation network is further discussed in the following section, particularly with respect to identifying mucosal sites that exhibit coordinated inflammatory, proliferative, and immunophenotypic remodeling without epithelial damage.

**Figure 6:**
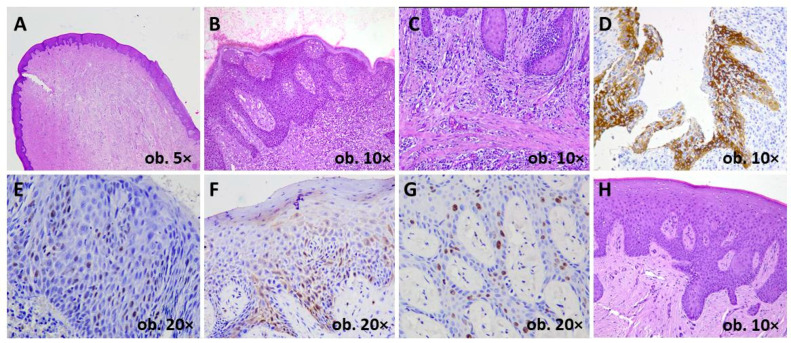
Representative histopathological and immunohistochemical findings of oral mucosal remodeling across study groups. Control mucosa showed preserved epithelial architecture and minimal inflammatory infiltrate on hematoxylin–eosin staining (**A**). In contrast, amalgam/metal-associated mucosa showed material-associated epithelial remodeling, with basal/parabasal hyperplasia, acanthosis, elongated epithelial ridges, and subepithelial inflammatory infiltrate (**B**). Material-associated mucosa also displayed subepithelial fibrosis, with stromal collagen condensation beneath the epithelium (**C**). Immunohistochemical analysis illustrated suprabasal CK19 redistribution (**D**), increased Ki67 labeling in the basal/parabasal proliferative compartment (**E**), moderate-to-strong epithelial and subepithelial COX-2 expression (**F**), and limited, heterogeneous nuclear p53 positivity (**G**). Ceramic-associated mucosa showed relatively preserved epithelial architecture and low-grade stromal remodeling on hematoxylin–eosin staining (**H**). Panels A–C and H show hematoxylin–eosin staining, whereas panels D–G show immunohistochemistry with DAB chromogen and hematoxylin counterstain. Original magnification: 10×; scale bars = 200 µm.

Representative histopathological and immunohistochemical findings are shown in Figure 6.

### 3.6. Multivariable Analysis

To evaluate whether the restorative material category remained independently associated with epithelial remodeling after adjustment for potential demographic confounders, multivariable regression analyses were performed using age and sex as covariates. The complete data is presented in Table 7.

In multivariable linear regression analysis, restorative material category remained independently associated with MAERS after adjustment for age and sex (*p* < 0.001). Metallic/metal-ceramic restorations demonstrated the strongest independent association with increased cumulative remodeling burden (β = 0.48, *p* < 0.001), followed by dental amalgam restorations (β = 0.44, *p* < 0.001). Resin composite-associated mucosa demonstrated a weaker but still statistically significant association with elevated MAERS values (β = 0.21, *p* = 0.018). In contrast, ceramic restorations did not show an independent association with increased remodeling burden compared with the control group after adjustment.

Binary logistic regression analyses additionally demonstrated significant independent associations between restorative material category and several high-grade remodeling outcomes. Metallic/metal-ceramic restorations were independently associated with high-grade inflammatory infiltrate (odds ratio [OR]: 5.84, 95% confidence interval [CI]: 2.11–16.17, *p* < 0.001), suprabasal CK19 redistribution (OR: 6.32, 95% CI: 2.28–17.49, *p* < 0.001), elevated Ki67 proliferative activity (OR: 5.97, 95% CI: 2.16–16.52, *p* < 0.001), and increased COX-2 expression (OR: 5.41, 95% CI: 1.98–14.78, *p* = 0.001).

Dental amalgam restorations demonstrated a similar independent remodeling profile, including significant associations with high-grade inflammatory infiltrate (OR = 5.12, 95% CI: 1.94–13.54, *p* = 0.001), suprabasal CK19 redistribution (OR = 5.76, 95% CI: 2.11–15.74, *p* < 0.001), elevated Ki67 proliferative activity (OR = 4.88, 95% CI: 1.86–12.81, *p* = 0.002), and increased COX-2 expression (OR = 4.63, 95% CI: 1.75–12.24, *p* = 0.002). Resin composite-associated mucosa demonstrated intermediate odds ratios for high-grade inflammatory infiltrate (OR = 2.47, 95% CI: 0.98–6.23, *p* = 0.056), high-grade CK19 redistribution (OR = 2.94, 95% CI: 1.12–7.69, *p* = 0.031), elevated Ki67 proliferative activity (OR = 2.51, 95% CI: 0.97–6.48, *p* = 0.059), and increased COX-2 expression (OR = 2.29, 95% CI: 0.88–5.95, *p* = 0.089). All-ceramic-associated specimens showed no statistically significant increase in remodeling risk for inflammatory infiltrate (OR = 1.18, 95% CI: 0.41–3.36, *p* = 0.761), CK19 redistribution (OR = 1.07, 95% CI: 0.36–3.12, *p* = 0.903), Ki67 proliferative activity (OR = 1.01, 95% CI: 0.34–2.98, *p* = 0.981), or COX-2 expression (OR = 0.96, 95% CI: 0.31–2.83, *p* = 0.944).

Age demonstrated weak positive associations with cumulative remodeling burden, whereas sex did not independently predict major histopathological or immunohistochemical remodeling outcomes in adjusted models.

Overall, multivariable analysis confirmed that restorative material category remained independently associated with cumulative epithelial remodeling burden and inflammation-associated epithelial plasticity after adjustment for demographic covariates. The strongest independent associations were consistently observed for metallic/metal-ceramic and dental amalgam restorations.

## 4. Discussion

### 4.1. Principal Findings

This retrospective comparative study demonstrated that oral mucosa adjacent to long-standing restorative materials exhibits distinct, material-associated epithelial and stromal remodeling profiles. The highest cumulative remodeling burden was observed in the metallic/metal–ceramic and dental amalgam groups, whereas ceramic-associated mucosa showed a profile similar to that of control specimens. Resin composite-associated mucosa displayed an intermediate pattern. Importantly, no epithelial dysplasia was identified in any group, indicating that the observed changes should be interpreted as non-dysplastic reactive remodeling rather than premalignant transformation.

### 4.2. Material-Associated Histopathological Remodeling

The metallic/metal–ceramic and dental amalgam groups showed the most pronounced histopathological remodeling, characterized by higher frequencies of basal hyperplasia, acanthosis, spongiosis, inflammatory infiltration, parakeratosis, and subepithelial fibrosis. From a histopathological perspective, basal hyperplasia reflects expansion of the basal/parabasal epithelial compartment, whereas acanthosis denotes thickening of the spinous epithelial layer. Spongiosis indicates intercellular edema in the epithelium and is commonly associated with inflammatory epithelial injury, while parakeratosis reflects altered keratin maturation, with persistence of nuclei in the keratinized layer. Inflammatory infiltration reflects the intensity of the local immune response in the lamina propria, and subepithelial fibrosis reflects chronic stromal remodeling characterized by increased collagen deposition beneath the epithelium. Taken together, these parameters describe a reactive mucosal remodeling pattern in which epithelial adaptation, inflammatory stimulation, and stromal response occur in parallel, without necessarily indicating epithelial dysplasia [5,6,7,8,9,10,20,21]. In contrast, ceramic-associated mucosa showed the lowest remodeling burden and remained close to the control group, while resin composite-associated specimens displayed an intermediate pattern. This distribution suggests that long-term mucosal adjacency to different restorative material categories is associated with unequal tissue adaptation rather than a uniform response to the mere presence of a restoration.

These findings are biologically plausible because restorative materials differ substantially in surface chemistry, corrosion behavior, ion release, roughness, plaque-retentive potential, and interaction with oral biofilms [1,2,3,22,23]. Metallic and metal–ceramic restorations may be particularly relevant in this context because cobalt–chromium and other dental alloys can release metal ions under oral conditions, especially when affected by surface modification, welding, corrosion, or local environmental changes [22]. This mechanism is consistent with recently proposed models linking corrosion-released metal ions from base-metal and porcelain-fused-to-metal restorations to activation of innate immune sensors and a downstream neuro-immune inflammatory cascade at the mucosal interface [24]. Such processes should not be interpreted as directly causing epithelial remodeling; rather, they may contribute to a local microenvironment in which material degradation products, plaque accumulation, and inflammatory mediators coexist at the material–mucosa interface [17,22,25].

From a clinical perspective, these findings do not support routine replacement of metallic, metal–ceramic, or dental amalgam restorations in asymptomatic patients. However, they suggest that long-standing restorations in close contact with oral mucosa should be carefully assessed when persistent erythema, lichenoid changes, discomfort, ulceration, plaque retention, marginal degradation, corrosion, roughness, or polishing defects are present [4,16,17,18,25]. In such cases, clinical management should include optimizing oral hygiene, controlling local plaque-retentive factors, smoothing or polishing rough surfaces when appropriate, and replacing the restoration only when there is a clear clinical indication or a persistent material-related mucosal reaction [1,2,3,16,17,18,23,25]. Therefore, the clinical relevance of this study lies in emphasizing that material-adjacent mucosa may exhibit non-dysplastic inflammatory remodeling and should be interpreted with careful clinicopathological correlation rather than solely on material type.

The higher inflammatory infiltrate and subepithelial fibrosis observed in the metallic/metal–ceramic and dental amalgam groups are consistent with previous clinical evidence on oral lichenoid contact lesions, dental metal allergy, type IV hypersensitivity reactions, and soft-tissue responses associated with restorative materials [4,16,17,18,25]. However, the present study differs from most previous reports in that it did not focus solely on clinically evident contact lesions or hypersensitivity reactions. Instead, it compared histologically preserved non-neoplastic oral mucosa across several restorative material categories and quantified remodeling even in the absence of epithelial dysplasia. This approach supports the interpretation that material-associated mucosal changes may exist along a spectrum ranging from subtle reactive remodeling to more clinically recognizable lichenoid or inflammatory lesions [8,9,10,20,21].

Previous clinical and histopathological studies indicate that oral mucosal responses to restorative and prosthetic materials are heterogeneous and depend on material type, local contact, and lesion context [4,16,17,18,19,25]. Reports on oral lichenoid contact lesions and material-associated mucosal reactions have linked dental amalgam and metallic restorations with localized inflammatory or interface changes, particularly when lesions are topographically related to the restoration [4,16,17,18,25]. Large patient cohorts further indicate that material-associated allergy, when confirmed by patch testing, most frequently involves metals and is associated with gingival and lichenoid changes adjacent to restorations, although the population-level prevalence of clinically confirmed amalgam-associated lichenoid lesions has been reported as low [26,27]. Consistent with these data, the present study found the highest remodeling burden in metallic/metal–ceramic and amalgam-associated mucosa, an intermediate profile in resin composite-associated specimens, and a comparatively low burden in ceramic-associated mucosa. This is consistent with clinical evidence that replacement of amalgam restorations with ceramic materials is associated with healing of adjacent lichenoid lesions, particularly when lesions are in close topographic contact with the restoration [28].

The comparatively low remodeling burden in all-ceramic-associated mucosa is also relevant. The similarity between the all-ceramic and control groups may be explained by the comparatively stable surface characteristics and lower degradation potential of all-ceramic restorations in comparison with metallic or amalgam-based restorations. In the present cohort, all-ceramic-associated mucosa showed low inflammatory scores, limited basal/parabasal expansion, reduced expression of CK19, Ki67, and COX-2, and MAERS values close to those of control mucosa. This pattern suggests that, under the conditions evaluated in this study, all-ceramic restorations were associated with a lower burden of chronic inflammatory epithelial remodeling. However, this finding should not be interpreted as absolute biological inertness, because ceramic surfaces may still be influenced by roughness, polishing quality, surface energy, aging, plaque accumulation, and local oral environmental conditions [1,3,23,29].

Resin composite-associated mucosa occupied an intermediate position between ceramic/control specimens and metallic/amalgam-associated specimens. This pattern may reflect the combined influence of surface roughness, plaque accumulation, resin matrix degradation, and local biofilm dynamics, all of which can affect the biological behavior of restorative surfaces in the oral cavity [1,2,3,23]. The intermediate histopathological scores suggest that resin composite restorations were associated with more remodeling than ceramic restorations, but less than metallic/metal–ceramic and dental amalgam restorations.

### 4.3. Immunohistochemical Evidence of Epithelial Plasticity

The coordinated increase in CK19 redistribution, Ki67 expression, and COX-2 immunoreactivity supports the presence of an inflammation-associated epithelial plasticity phenotype rather than isolated marker variation [11,12,13,30]. In the present cohort, this pattern was most evident in metallic/metal–ceramic and dental amalgam-associated mucosa, less pronounced in resin composite specimens, and minimal in ceramic and control groups, paralleling the histopathological remodeling gradient observed across material categories.

The higher CK19, Ki67, and COX-2 values observed in the metallic/metal–ceramic and dental amalgam groups may be explained by the combined influence of chronic local inflammation, plaque-retentive surface characteristics, corrosion or degradation products, and possible metal ion release at the material–mucosa interface [16,17,18,22,25,30]. In this microenvironment, inflammatory mediators may promote COX-2 expression, while persistent epithelial stimulation may increase basal/parabasal proliferative activity, reflected by Ki67, and alter epithelial differentiation patterns, reflected by suprabasal CK19 redistribution [11,12,13]. This proliferative and inflammatory marker profile parallels findings in amalgam-associated lichenoid lesions, where Ki-67 proliferation indices have been reported in a comparable range [31,32]. Therefore, the increased immunohistochemical expression observed in these groups is best interpreted as part of a coordinated, non-dysplastic inflammatory remodeling response rather than as evidence of malignant transformation.

Suprabasal CK19 expression may indicate altered epithelial differentiation or expansion of a less mature epithelial compartment [11,12]. Increased Ki67 expression reflects enlargement of the proliferative basal/parabasal cell population and supports the interpretation of epithelial compartment expansion in reactive or inflammatory oral mucosal contexts [11,12,13,14]. COX-2 expression is closely related to inflammatory signaling and may connect stromal inflammatory activity with epithelial response patterns [12,13]. In the present study, the parallel increases in CK19, Ki67, and COX-2 suggest that mucosal remodeling adjacent to metallic/metal–ceramic and dental amalgam restorations involved both epithelial proliferative adaptation and activation of the inflammatory pathway.

These findings are consistent with previous evidence indicating that long-term contact with dental amalgam and other restorative materials may be associated with histopathological and immunohistochemical epithelial responses, particularly in inflammatory, reactive, or contact-related mucosal settings [11,13,16,17,18,30]. However, the present study extends this perspective by comparing multiple restorative material categories within the same histopathological and immunohistochemical framework. The lower CK19, Ki67, and COX-2 scores in ceramic-associated mucosa support the comparatively stable histopathological profile observed in this group, whereas resin composite specimens showed an intermediate immunophenotypic pattern.

Importantly, this CK19–Ki67–COX-2 axis should not be interpreted as evidence of dysplastic transformation. Rather, in the absence of architectural or cytological dysplasia, it supports a non-dysplastic remodeling phenotype associated with chronic local inflammatory adaptation [9,11,12,15].

### 4.4. Interpretation of p53 Expression

p53 expression requires careful interpretation in the context of the present study. Although p53 showed statistically significant intergroup variation, its distribution was less structured than CK19, Ki67, and COX-2, and strong diffuse overexpression was not observed as a dominant pattern in any restorative material group. This finding suggests that p53 was not part of the coordinated CK19–Ki67–COX-2 remodeling axis identified in this cohort.

In non-dysplastic oral mucosa, limited or heterogeneous p53 nuclear staining should not be interpreted as evidence of malignant transformation or TP53 mutational status. Instead, it may reflect a variable cellular stress response occurring in reactive or inflammation-associated epithelial settings [11,13,15,32]. This interpretation is supported by the absence of architectural or cytological epithelial dysplasia in all study groups and by the weaker correlations between p53 expression and the main remodeling parameters.

This distinction is clinically relevant because reactive, lichenoid, and dysplastic epithelial changes may overlap morphologically and require careful clinicopathological correlation [8,9,10,20,21]. In this study, p53 expression should be considered more of a secondary stress-related marker rather than a primary indicator of material-associated epithelial plasticity or malignant potential. This interpretation is supported by recent comparative data showing that p53, Ki-67, and COX-2 expression in lichenoid-pattern oral mucosa did not differ significantly from healthy controls, whereas p63 was selectively decreased, suggesting that these markers reflect a non-specific stress and inflammatory response rather than a dysplasia-associated signature [33]. This is further supported by meta-analytic evidence that Ki-67 expression is significantly lower in non-dysplastic lichenoid oral mucosa than in oral epithelial dysplasia, suggesting that the moderate, non-dysplastic Ki-67 elevation observed in the present cohort reflects reactive epithelial plasticity rather than premalignant proliferative activity [34].

### 4.5. Relevance of MAERS and Adjusted Analysis

The Material-Associated Epithelial Remodeling Score (MAERS) was designed to integrate histopathological inflammation, basal epithelial expansion, CK19 redistribution, Ki67 proliferative activity, and COX-2 expression into a single cumulative measure of epithelial and stromal remodeling. The inclusion of these parameters is biologically justified because inflammatory infiltrate, epithelial compartment expansion, altered CK19 distribution, increased Ki67 activity, and COX-2 expression reflect interconnected components of inflammation-associated epithelial plasticity [5,6,7,11,12,13].

In the present study, MAERS showed a clear material-associated gradient, with the highest values in metallic/metal–ceramic and dental amalgam groups, intermediate values in resin composite specimens, and the lowest values in ceramic and control groups. This distribution supports the interpretation that cumulative remodeling burden differs across restorative material categories rather than being uniformly related to the presence of a restoration.

The strong correlations between MAERS and inflammatory infiltrate, CK19 redistribution, Ki67 proliferative activity, and COX-2 expression indicate that the score captured a coordinated remodeling phenotype rather than isolated histological or immunohistochemical variation. This is consistent with the concept that epithelial turnover, immunophenotypic redistribution, stromal inflammation, and inflammatory signaling are biologically linked in reactive oral mucosal adaptation [5,6,11,12,13].

From a clinical perspective, the correlation between inflammatory infiltrate, CK19 redistribution, Ki67 proliferative activity, COX-2 expression, and MAERS suggests that material-adjacent mucosa may undergo a coordinated remodeling response rather than isolated microscopic changes. This may be relevant to identifying mucosal sites where chronic local irritation, plaque accumulation, material degradation, or restoration-related factors are associated with increased epithelial turnover and inflammatory signaling. Although these findings do not indicate epithelial dysplasia or malignant transformation, they support careful clinical monitoring of mucosal areas adjacent to long-standing restorations, particularly when persistent erythema, lichenoid changes, discomfort, plaque retention, or surface deterioration are present.

Multivariable analysis further supported an independent association between restorative material category and remodeling burden after adjustment for age and sex. Metallic/metal–ceramic and dental amalgam restorations showed the strongest adjusted associations with increased MAERS and high-grade remodeling outcomes. In contrast, ceramic restorations did not show an independent association with increased remodeling burden compared with control mucosa.

Therefore, MAERS may be useful as an exploratory composite measure for summarizing material-associated epithelial remodeling in retrospective histopathological cohorts. However, because MAERS was developed for this study, it should be considered a research tool rather than a validated clinical index until it is externally validated in independent cohorts.

### 4.6. Limitations and Future Perspectives

This study has several limitations that should be acknowledged. First, its retrospective design prevents causal inference; therefore, the observed differences should be interpreted as associations between restorative material category and mucosal remodeling, not as evidence that specific materials directly cause epithelial or stromal changes. Consistent with the data availability constraints noted in Section 2.3, several patient- and material-level variables could not be systematically assessed across all cases, including periodontal status, oral hygiene, plaque accumulation, occlusal trauma, restoration age or surface characteristics (roughness, corrosion, polishing quality, marginal degradation), local microbiota, smoking status, alcohol consumption, diabetes mellitus, systemic inflammatory background, and medication use. Although groups were balanced for major clinicodemographic variables, and cases with multiple adjacent restorative materials were excluded to reduce exposure misclassification, residual confounding from these unmeasured factors cannot be fully excluded.

Second, material exposure was defined according to clinical documentation and long-standing mucosal adjacency, but no direct physicochemical analysis of the restorative surfaces was performed. Therefore, surface roughness, corrosion, ion release, resin degradation, and biofilm composition could not be directly correlated with tissue-level remodeling. Third, the study included archived formalin-fixed, paraffin-embedded specimens obtained during alveoloplastic extraction procedures; consequently, the results may not be fully generalizable to all oral mucosal sites or to clinically symptomatic contact lesions. Fourth, MAERS was constructed specifically for the present study and should therefore be interpreted as an exploratory composite score designed to summarize cumulative epithelial remodeling burden. Although its internal consistency was good, external validation in independent cohorts is required before broader clinical or research application.

Future prospective studies should integrate standardized clinical exposure assessment, detailed restoration characterization, surface analysis, microbiological profiling, patient-level inflammatory and behavioral risk factors, and longer follow-up. Such studies may clarify whether the material-associated remodeling patterns observed here remain stable, regress after removal or replacement of the adjacent restoration, or progress toward clinically recognizable inflammatory or lichenoid mucosal conditions.

## 5. Conclusions

This retrospective histopathological and immunohistochemical study showed that long-standing mucosal adjacency to different restorative material classes is associated with distinct, non-dysplastic epithelial and stromal remodeling profiles. Metallic/metal–ceramic and dental amalgam restorations showed the highest remodeling burden, resin composite restorations showed an intermediate profile, and ceramic-associated mucosa remained close to that of control specimens.

The coordinated increase in inflammatory infiltrate, CK19 redistribution, Ki67 proliferative activity, and COX-2 expression supports an inflammation-associated epithelial plasticity phenotype, particularly in metallic/metal–ceramic and amalgam-associated mucosa. In contrast, p53 expression should be interpreted cautiously as a heterogeneous cellular stress-response marker rather than as evidence of malignant transformation in the absence of epithelial dysplasia.

MAERS may serve as an exploratory composite measure of material-associated mucosal remodeling, but further validation is required before broader clinical or research use. Future studies should include prospective multicenter cohorts, independent external validation, longitudinal follow-up, correlation with clinical and restoration-related variables, and assessment of reproducibility and clinically meaningful threshold values.

## Figures and Tables

**Table 1:** Semi-Quantitative Grading Criteria for Histopathological Parameters.

Histopathological Parameter	Score Range	Grading Criteria
Basal hyperplasia	0–3	The basal layer expansion and ridge elongation
Acanthosis	0–3	Spinous layer thickening
Parakeratosis	0–1	Absence/presence of parakeratosis
Spongiosis	0–3	The extent of intercellular edema
Subepithelial fibrosis	0–3	The density of collagen deposition beneath the epithelium
Inflammatory infiltrate	1–3	Intensity and distribution of the inflammatory infiltrate

**Table 2:** Primary antibodies used.

Marker	Clone	Host/Isotype	Retrieval Buffer	Supplier
CK19	RCK108	mouse monoclonal	Citrate pH 6.0	Agilent Technologies, Santa Clara, CA, USA
Ki67	MIB-1	mouse monoclonal	Citrate pH 6.0	Dako, Glostrup, Denmark
p53	DO-7	mouse monoclonal	EDTA pH 9.0	Dako, Glostrup, Denmark
COX-2	SP21	rabbit monoclonal	Citrate pH 6.0	Thermo Fisher Scientific, Waltham, MA, USA

Dilutions followed the manufacturer’s specifications for each antibody.

**Table 3:** Immunohistochemical Scoring Criteria.

Marker	Score Range	Scoring Criteria
CK19	0–3	0—no staining
		1—staining confined to basal/parabasal layers
		2—extension into suprabasal layers
		3—diffuse suprabasal or near full-thickness positivity
Ki67	0–2	0—≤5% positive nuclei
		1—6–10% positive nuclei
		2—≥11% positive nuclei
p53	0–2	0—<5% positive cells
		1—6–10% positive cells
		2—≥11% positive cells with moderate-to-strong intensity
COX-2	0–3	0—no staining
		1—weak focal staining
		2—moderate multifocal staining
		3—strong diffuse staining

**Table 4:** Clinicodemographic Characteristics of the Study Cohort.

Parameter	Control (*n* = 30)	Amalgam (*n* = 30)	Resin Composite (*n* = 30)	Ceramic (*n* = 30)	Metallic/Metal-Ceramic (*n* = 30)	*p*-Value
Age, years, mean ± SD	49.2 ± 8.7	51.6 ± 9.1	48.8 ± 8.4	50.3 ± 7.9	52.1 ± 8.8	0.412
Female, *n* (%)	17 (56.7)	18 (60.0)	16 (53.3)	17 (56.7)	18 (60.0)	0.962
Male, *n* (%)	13 (43.3)	12 (40.0)	14 (46.7)	13 (43.3)	12 (40.0)	0.962
Gingival mucosa, *n* (%)	19 (63.3)	20 (66.7)	18 (60.0)	19 (63.3)	20 (66.7)	0.973
Alveolar mucosa, *n* (%)	11 (36.7)	10 (33.3)	12 (40.0)	11 (36.7)	10 (33.3)	0.973
Contact duration, years, mean ± SD	NA	12.8 ± 5.4	11.9 ± 4.8	10.7 ± 4.2	13.4 ± 5.7	0.286
Contact duration ≥5 years, *n* (%)	NA	30 (100.0)	30 (100.0)	30 (100.0)	30 (100.0)	NA

Note: SD = standard deviation; NA = not applicable. All specimens were obtained during alveoloplastic extraction procedures. Contact duration refers only to exposed groups.

**Table 5:** Distribution of Histopathological Parameters According to Restorative Material Category.

Histopathological Parameter	Score	Control (*n* = 30)	Amalgam (*n* = 30)	Resin Composite (*n* = 30)	Ceramic (*n* = 30)	Metallic/Metal-Ceramic (*n* = 30)	*p*-Value
Basal hyperplasia	0	6	2	4	7	1	<0.001
	1	18	9	14	17	8	
	2	6	14	10	5	14	
	3	0	5	2	1	7	
Acanthosis	0	1	0	1	2	0	0.002
	1	20	8	14	19	7	
	2	9	16	12	8	15	
	3	0	6	3	1	8	
Parakeratosis	0	27	16	20	25	14	0.004
	1	3	14	10	5	16	
Spongiosis	0	14	5	8	13	4	0.006
	1	11	10	12	12	9	
	2	5	11	8	4	11	
	3	0	4	2	1	6	
Subepithelial fibrosis	0	4	1	3	5	1	<0.001
	1	18	8	14	17	7	
	2	8	15	11	7	14	
	3	0	6	2	1	8	
Inflammatory infiltrate	1	22	8	14	21	6	<0.001
	2	8	15	12	8	14	
	3	0	7	4	1	10	

Note: Histopathological scores were compared among groups using the Kruskal–Wallis test. The *p*-value refers to the overall intergroup comparison for each histopathological parameter, not to each score category. Basal hyperplasia, acanthosis, spongiosis, and subepithelial fibrosis were graded on a scale of 0 to 3. Parakeratosis was recorded as absent (0) or present (1). The inflammatory infiltrate was graded as 1 (mild and focal), 2 (moderate and diffuse), or 3 (intense). No epithelial dysplasia was identified in any study group.

**Table 6:** Distribution of Immunohistochemical Markers According to Restorative Material Category.

Immunohistochemical Marker	Score	Control (*n* = 30)	Amalgam (*n* = 30)	Resin Composite (*n* = 30)	Ceramic (*n* = 30)	Metallic/Metal-Ceramic (*n* = 30)	*p*-Value
CK19	0	9	2	5	8	1	<0.001
	1	16	8	13	17	7	
	2	4	13	9	4	13	
	3	1	7	3	1	9	
Ki67	0	8	2	5	9	1	<0.001
	1	18	9	14	17	7	
	2	4	19	11	4	22	
p53	0	21	15	18	22	13	0.031
	1	7	10	8	6	10	
	2	2	5	4	2	7	
COX-2	0	12	3	7	13	2	<0.001
	1	14	8	13	13	7	
	2	4	12	8	3	12	
	3	0	7	2	1	9	

Note: Immunohistochemical scores were compared among groups using the Kruskal–Wallis test. The *p*-value refers to the overall intergroup comparison for each immunohistochemical marker, not to each score category. CK19 was scored from 0 to 3 according to epithelial distribution and staining intensity. Ki-67 was scored from 0 to 2 based on the percentage of positive epithelial nuclei. p53 was scored from 0 to 2 based on nuclear staining extent and intensity. COX-2 was scored from 0 to 3 according to epithelial and subepithelial staining intensity and distribution. For each marker and group, values represent the number of cases assigned to each score category. Strong diffuse p53 overexpression was not observed as a dominant pattern in any group.

**Table 7:** Age- and Sex-Adjusted Regression Analysis of Remodeling Outcomes.

Outcome Variable	Predictor	Adjusted β/OR	95% CI	*p*-Value
MAERS (linear regression)	Metallic/metal-ceramic restorations	β = 0.48	0.31–0.65	<0.001
	Dental amalgam restorations	β = 0.44	0.27–0.61	<0.001
	Resin composite restorations	β = 0.21	0.04–0.38	0.018
	Ceramic restorations	β = 0.06	−0.09–0.21	0.412
High-grade inflammatory infiltrate (≥2)	Metallic/metal-ceramic restorations	OR = 5.84	2.11–16.17	<0.001
	Dental amalgam restorations	OR = 5.12	1.94–13.54	0.001
	Resin composite restorations	OR = 2.47	0.98–6.23	0.056
	Ceramic restorations	OR = 1.18	0.41–3.36	0.761
High-grade CK19 redistribution (≥2)	Metallic/metal-ceramic restorations	OR = 6.32	2.28–17.49	<0.001
	Dental amalgam restorations	OR = 5.76	2.11–15.74	<0.001
	Resin composite restorations	OR = 2.94	1.12–7.69	0.031
	Ceramic restorations	OR = 1.07	0.36–3.12	0.903
Elevated Ki67 proliferative activity (score 2)	Metallic/metal-ceramic restorations	OR = 5.97	2.16–16.52	<0.001
	Dental amalgam restorations	OR = 4.88	1.86–12.81	0.002
	Resin composite restorations	OR = 2.51	0.97–6.48	0.059
	Ceramic restorations	OR = 1.01	0.34–2.98	0.981
Increased COX-2 expression (≥2)	Metallic/metal-ceramic restorations	OR = 5.41	1.98–14.78	0.001
	Dental amalgam restorations	OR = 4.63	1.75–12.24	0.002
	Resin composite restorations	OR = 2.29	0.88–5.95	0.089
	Ceramic restorations	OR = 0.96	0.31–2.83	0.944

Note: Multivariable linear regression was used for continuous MAERS analysis, whereas binary logistic regression was used for high-grade remodeling outcomes. All models were adjusted for age and sex; these covariates were included but are not displayed as separate predictors in the table to maintain readability. Control specimens served as the reference category for comparisons of restorative materials. β = standardized regression coefficient; OR = odds ratio; CI = confidence interval.

## Data Availability

Data supporting the reported results are available from the corresponding authors upon reasonable request.
